# Flavonoid Interactions with Renal Organic Anion Transporters OAT1 and OAT3: Structure–Activity Relationships and Clinical Implications

**DOI:** 10.3390/ijms27073310

**Published:** 2026-04-06

**Authors:** Kai Tong, Pinmao Ye, Kazuko Kaneda-Nakashima, Han Zhang, Ling Wei

**Affiliations:** 1 School of Chinese Materia Medica, Guangdong Pharmaceutical University, Guangzhou 510006, China; 13118762620@163.com (K.T.); pinmao13@163.com (P.Y.); 2 Institute for Radiation Sciences, The University of Osaka, 2-4 Yamadaoka, Suita 565-0871, Japan; kanedak17@chem.sci.osaka-u.ac.jp; 3 Core for Medicine and Science Collaborative Research and Education, Forefront Research Center, Graduate School of Science, The University of Osaka, 1-1 Machikaneyama, Toyonaka 560-0043, Japan

**Keywords:** flavonoids, organic anion transporters (OAT1/OAT3), phase II metabolites, structure–activity relationship (SAR), herb–drug interactions, nephroprotection

## Abstract

Renal organic anion transporters 1 (OAT1) and 3 (OAT3) mediate the excretion of endogenous metabolites and xenobiotics. Flavonoids interact significantly with these transporters, but the structural determinants—especially regarding in vivo phase II metabolism—remain unclear. This review integrates recent cryogenic electron microscopy (cryo-EM) structural biology and transporter kinetics to delineate the molecular basis of flavonoid–OAT interactions. We highlight phase II metabolites as key in vivo effectors. Structurally, OAT1 strictly favors compact, planar anionic scaffolds, whereas OAT3 accommodates bulkier, conjugated forms. Crucially, flavonoids exert a “double-edged” toxicological effect: high-affinity OAT inhibition risks herb–drug interactions, yet competitively limits the tubular uptake of nephrotoxins. Furthermore, disease states and post-translational regulation reshape these interactions. By bridging structural insights with biomarker-guided pharmacokinetics, we propose a mechanistic framework to improve the precise safety assessment of flavonoid-containing therapeutics.

## 1. Introduction

Flavonoids, a diverse group of naturally occurring polyphenols widely distributed in dietary and medicinal plants, have attracted substantial attention for their broad biomedical activities, including anti-inflammatory, antioxidant, anticancer, and cardioprotective effects. Recent advances further highlight their therapeutic potential in combination treatment strategies, where flavonoids have been shown to overcome multidrug resistance and synergistically enhance the efficacy of conventional therapeutics, while innovative nanoformulations have been developed to improve their bioavailability [1,2]. These pharmacological effects are fundamentally governed by specific structural features of flavonoids, such as hydroxylation, methylation, and glycosylation patterns, which dictate their molecular interactions and biological activity [1,2]. Beyond target-site efficacy, these same structural determinants decisively govern how flavonoids interact with key pharmacokinetic proteins in vivo, including renal drug transporters.

Organic anion transporters 1 (OAT1) and 3 (OAT3) are predominantly localized on the basolateral membrane of proximal tubule epithelial cells. They serve as critical rate-limiting transporters that mediate the renal excretion of diverse endogenous metabolites (e.g., uremic toxins) and exogenous drugs (e.g., β-lactam antibiotics) [3,4,5]. Beyond this classical excretory function, the “Remote Sensing and Signaling” theory proposed by Nigam et al. has redefined OAT1/OAT3 as key components linking the gut–liver–kidney metabolic axis. By orchestrating the inter-organ flux of small molecules, these transporters contribute to the regulation of systemic metabolic homeostasis [3,4]. Importantly, their functional capacity is not static but exhibits substantial physiological variability. OAT1/OAT3 expression is influenced by developmental stage (ontogeny), sexual dimorphism, and complex signaling networks involving protein kinase C (PKC) and nuclear factor erythroid 2-related factor 2 (Nrf2) pathways [6,7,8]. Such heterogeneity implies that renal handling of substrates may differ across pediatric, geriatric, or sex-specific populations, thereby influencing the safety profile of co-administered therapeutics.

Given their polyspecific nature, OATs are susceptible to modulation by diverse dietary and herbal constituents, among which flavonoids represent an important class of natural OAT1/OAT3 modulators [3,9]. However, a critical pharmacokinetic complexity lies in their biotransformation: unlike the parent aglycones typically evaluated in in vitro assays, flavonoids circulate in vivo primarily as phase II metabolites (e.g., glucuronides and sulfates) [8,9]. These anionic conjugates often display transporter affinities distinct from their parent compounds, giving rise to a clinically relevant “double-edged” effect. High-affinity metabolites may competitively inhibit OAT-mediated transport, potentially retaining narrow-therapeutic-index drugs (e.g., carbapenems, methotrexate) and increasing the risk of herb–drug interactions (HDI) [9,10,11]; conversely, such competitive interactions may reduce renal tubular uptake of nephrotoxic substrates such as cisplatin, thereby conferring nephroprotective potential [12,13,14].

Understanding this regulatory complexity requires a systematic analysis of the structural determinants governing flavonoid–OAT interactions. To this end, this review examines these interactions from a structure–activity relationship (SAR) perspective. We highlight the roles of the B-ring catechol moiety, planar conjugated systems, and phase II metabolic modifications in modulating transporter affinity and subtype selectivity. We further discuss how these structural insights may inform herb–drug interaction risk prediction and the rational evaluation of nephroprotective strategies.

## 2. Structural and Functional Overview of Renal OAT1/OAT3

### 2.1. Molecular Architecture and Transport Mechanisms

Renal organic anion transporters 1 (OAT1) and 3 (OAT3), encoded by the *SLC22A6* and *SLC22A8* genes, are members of the solute carrier 22 (SLC22) family. They share the canonical major facilitator superfamily (MFS) fold. This architecture features a conserved 12-transmembrane (TM) helix topology and a large glycosylated extracellular loop, structural hallmarks for proper membrane trafficking and protein stability [4,15,16,17,18].

Recent advances in structural biology have elucidated the substrate recognition mechanism of human OAT1 at atomic resolution. In 2025, Zhang et al. and Jeon et al. independently captured high-resolution cryogenic electron microscopy (cryo-EM) structures of OAT1 in ligand-free (apo), substrate-bound, and inhibitor-bound states. These structures revealed that a central cavity formed by TM1, TM4, TM7, and TM10 functions to “clamp” the ligand, with positively charged residues guiding substrate orientation via electrostatic steering [17,18]. Collectively, these findings structurally validate the alternating-access transport cycle and provide a mechanistic framework for interpreting the structure–activity relationships (SAR) of flavonoids with OAT1 [17,18,19].

In contrast, although a high-resolution structure for OAT3 remains elusive, homology modeling and mutagenesis studies indicate that OAT3 possesses a larger and more hydrophobic binding pocket than OAT1. The key residue Arg454 synergizes with the surrounding hydrophobic network to accommodate bulky anionic substrates, including flavonoid glucuronides [20,21]. This structural divergence underpins the broader substrate promiscuity of OAT3, offering a mechanistic rationale for its preferential recognition of Phase II flavonoid conjugates [17,18,19].

### 2.2. Tissue Distribution and Renal Localization

Both OAT1 and OAT3 are abundantly expressed at the basolateral membrane of the renal proximal tubule in rodents and humans, functioning as the primary gateways for the organic anion influx from blood into tubular epithelial cells. Beyond the kidney, their expression in the choroid plexus, spinal cord, placenta, and the eye suggests a broader physiological role in maintaining local barrier integrity and organ protection [6,7,22,23].

Immunohistochemical mapping by Breljak et al. revealed that murine Oat1/Oat3 were predominantly localized to the S2–S3 segments of the proximal tubule, with weaker expression in the S1 segment. Notably, their expression profiles exhibit significant sex-dependent and ontogenic heterogeneity: adult males show higher expression levels than females, and transporter abundance increases progressively from the neonatal period through weaning. This developmental trajectory aligns with established ontogenetic patterns where OAT1/OAT3 levels are low during embryogenesis, rise postnatally, and plateau at adult levels post-puberty. This implies that pediatric populations may exhibit OAT-mediated drug exposure risks and nephrotoxicity profiles that differ significantly from those in adults [7].

Consistent with findings in animal models, OAT1 and OAT3 in the human kidney are predominantly localized to the basolateral membrane of the cortical proximal tubule, serving as the major entry pathways for anionic drugs and uremic toxins. Using conditionally immortalized human proximal tubule epithelial cells (ciPTEC), Nieskens et al. demonstrated that OAT1 overexpression significantly exacerbated the cytotoxicity of the antiviral drug cidofovir, thereby identifying OAT-mediated uptake as a rate-limiting step in tubular injury [24]. Collectively, previous studies indicate that the high renal abundance of OAT1/OAT3 constitutes the critical anatomical basis for their involvement in both drug-associated nephrotoxicity and nephroprotective effects [22,25,26].

### 2.3. Transport Mechanism and Substrate Spectrum

OAT1/OAT3-mediated transport is not simple facilitated diffusion but a classic “tertiary active transport” system driven by metabolic energy. Mechanistically, the transmembrane Na^+^ gradient established by Na^+^/K^+^-ATPase drives the Na^+^-dicarboxylate cotransporter 3 (NaDC3) to concentrate dicarboxylates (e.g., α-ketoglutarate, α-KG) intracellularly. OATs then utilize this high intracellular α-KG gradient as the driving force to exchange organic anions against their concentration gradient, coupling drug uptake with α-KG efflux [3,22].

As elucidated by recent high-resolution cryo-EM structures of human OAT1 (2025), this transporter adopts the canonical Major Facilitator Superfamily (MFS) fold with 12 transmembrane helices and operates via an “alternating access” mechanism, cycling through outward-open, occluded, and inward-open conformations. The substrate-binding pocket, defined by an aromatic hydrophobic cage and electrostatic anchor points, underlies the remarkable polyspecificity of OAT1. Competitive binding between organic anion substrates and the endogenous dicarboxylate *α*-ketoglutarate drives conformational transitions, enabling the precise coupling of substrate uptake with *α*-KG efflux [17,18].

Despite sharing overlapping substrates, OAT1 and OAT3 exhibit distinct recognition preferences. Specifically, OAT1 displays high affinity for smaller, hydrophilic anions (e.g., p-aminohippurate, adefovir, furosemide, methotrexate). In contrast, OAT3 is more structurally promiscuous, readily accommodating bulky, amphiphilic anions and conjugates, including nonsteroidal anti-inflammatory drugs (NSAIDs), enalaprilat, and flavonoid glucuronides [5,9].

Beyond xenobiotics, OATs govern the excretion of key endogenous metabolites, such as uric acid, indoxyl sulfate, hippuric acid, and prostaglandins [15,22,23,27]. Based on this, Nigam et al. proposed that by regulating the dynamic homeostasis of these metabolites, OAT1/OAT3 orchestrate a “Remote Sensing and Signaling” network that connects the kidney with the heart, brain, and gut [15]. Recently, population pharmacokinetic studies by Thakur et al. confirmed that the clearance of pyridoxic acid correlates strongly with OAT activity. This establishes pyridoxic acid as a sensitive endogenous biomarker for assessing in vivo OAT function, offering a novel tool for predicting drug–drug interactions (DDI) without exogenous probes [27].

**Figure 1:**
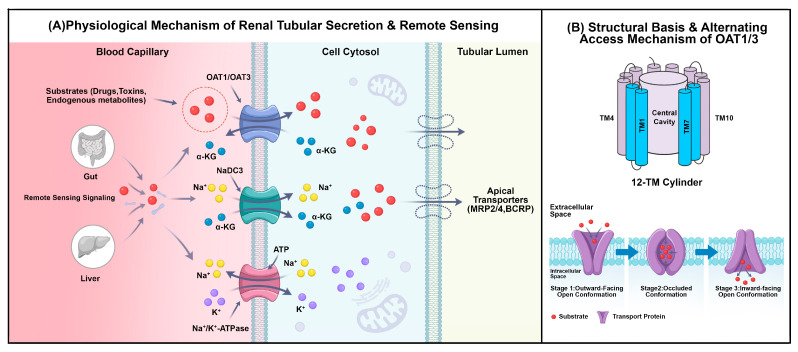
Schematic diagram of the tertiary active transport mechanism of OAT1/OAT3 in the renal proximal tubule and the “Remote Sensing and Signaling” model. (OAT1/3, organic anion transporter 1/3; α-KG, α-ketoglutarate; NaDC3, Na^+^-dicarboxylate cotransporter 3; MRP2/4, multidrug resistance-associated proteins 2/4; BCRP, breast cancer resistance protein).

In summary, by virtue of their polyspecificity, OAT1/OAT3 tightly couple the excretion of xenobiotics with endogenous metabolic flux, constituting a pivotal hub linking pharmacokinetics to systemic homeostasis (Figure 1A for the physiological mechanism, and Figure 1B for the structural alternating access). This physiological duality provides the theoretical premise for the following analysis of flavonoid-OAT interactions.

### 2.4. Physiological and Pathological Functions and Expression Regulation

Under physiological conditions, OAT1/OAT3 constitute the initial step of renal tubular secretion. Tightly coupled with apical efflux transporters such as multidrug resistance-associated proteins 2/4 (MRP2/4) and breast cancer resistance protein (BCRP), they form an efficient vectorial transport system that governs the renal clearance and therapeutic efficacy of anionic drugs, including diuretics (e.g., furosemide, thiazides) [28]. Beyond drug disposition, this system contributes to the regulation of systemic acid–base balance, vascular function, and the gut–microbiome–host metabolic axis by modulating the levels of uremic toxins, metabolic signaling molecules, and redox-active small metabolites [22,29,30].

Under pathological conditions, however, adaptive alterations in the expression and function of OAT1/OAT3 frequently occur. Accumulating evidence, including recent findings by You et al. (2025), indicates that both transcriptional and protein levels of OAT1/OAT3 are markedly downregulated during the progression of chronic kidney disease (CKD), acute kidney injury (AKI), and renal interstitial fibrosis [23]. This loss of transport capacity compromises substrate clearance and promotes the accumulation of uremic toxins and inflammatory metabolites, driving a vicious cycle of impaired transport, toxin retention, and progressive tissue injury [23,29]. Of note, OAT1/OAT3 also serve as critical gateways mediating drug-induced nephrotoxicity. Nephrotoxic agents such as antivirals (e.g., cidofovir) and methotrexate rely on OAT-mediated uptake to enter proximal tubular cells, resulting in intracellular accumulation and cytotoxicity. In contrast, pharmacological inhibition of these transporters by probenecid can significantly attenuate such renal injury [16].

The expression and activity of OAT1/OAT3 are subject to fine-tuned regulation by multi-level signaling networks. At the transcriptional level, pathways involving hepatocyte nuclear factors (HNFs), peroxisome proliferator-activated receptors (PPARs), Nrf2, and sex hormone signaling play central roles [7,31]. At the post-translational level, the PKC–Nedd4-2 axis represents the most well-characterized mechanism for rapid regulation: PKC activation enhances Nedd4-2-mediated ubiquitination and lysosomal degradation of OATs, leading to acute suppression of transporter activity [32,33].

Accumulating evidence suggests that clinical drugs and pathological factors modulate OAT function through these signaling networks, thereby influencing nephrotoxicity risks [25,26]. Collectively, these regulatory features suggest that flavonoids may modulate OAT1/OAT3 function through two distinct mechanisms: direct competitive interaction driven by substrate structure, and indirect regulation of transporter expression via upstream signaling pathways such as PKC and Nrf2. This dual regulatory framework provides the theoretical foundation for the subsequent SAR-based analysis of flavonoid–OAT interactions.

## 3. Structure–Activity Relationship (SAR) of Flavonoids

### 3.1. Basic Skeleton and Structural Classification

Flavonoids represent a diverse class of polyphenolic secondary metabolites widely distributed in dietary and medicinal plants, with over 8000 individual compounds identified to date [34]. Structurally, they share a characteristic C6–C3–C6 backbone composed of two benzene rings (rings A and B) linked by an oxygen-containing heterocyclic ring (ring C). With the exception of isoflavones, most naturally occurring flavonoids can be regarded as derivatives of 2-phenylchromen-4-one, a core scaffold characterized by pronounced planarity and conjugation [35]. Based on variations in the oxidation state and saturation of the C-ring, as well as the attachment position of the B-ring, flavonoids are classified into several subclasses [35]. Among those most extensively investigated for interactions with renal OAT1/OAT3, flavones (e.g., apigenin, luteolin) possess a C2=C3 double bond and a 4-oxo group on the C-ring; flavonols (e.g., quercetin, kaempferol) are distinguished by an additional 3-hydroxyl group (3-OH); flavan-3-ols (e.g., catechin) feature a saturated C-ring with a 3-OH substituent; and isoflavones (e.g., daidzein, genistein) differ by a shift in the position of the B-ring attachment from C2 to C3 [34,35].

In plants, these core skeletons are frequently modified through hydroxylation, methylation, acylation, and glycosylation, generating extensive structural diversity. Previous reviews have emphasized that the redox state of the C-ring, substitution patterns on the B-ring, and the sites of glycosylation collectively constitute critical structural determinants underlying the broad pharmacological activities of flavonoids, including anti-inflammatory, antitumor, and cardiovascular protective effects [36,37,38].

Notably, although flavonoids predominantly occur as O-glycosides or C-glycosides in plants, their chemical forms and bioavailability undergo profound transformation in humans. After intestinal hydrolysis of dietary glycosides, the liberated aglycones are absorbed and rapidly subjected to extensive Phase II metabolism in the liver and small intestine, yielding predominantly glucuronide and sulfate conjugates [39,40,41]. This biotransformation markedly increases molecular polarity and confers a negative charge, rendering flavonoid metabolites structurally well aligned with the canonical substrate profile of OAT1 and OAT3 [40]. Consequently, these metabolic features provide the physicochemical foundation for the subsequent SAR analysis, which focuses on the interactions of flavonoid aglycones and their Phase II conjugates with renal OATs.

### 3.2. Key Structural Features and the Physicochemical Basis of Interactions with OATs

Extensive structure–activity relationship (SAR) analyses and molecular docking studies indicate that the recognition and transport of flavonoids by OAT1 and OAT3 rely on a delicate balance between a hydrophobic aromatic scaffold and specific charge distributions [9,11]. Three key structural features collectively constitute the physicochemical basis underlying these interactions.

One fundamental determinant of transporter affinity is the molecular planarity of the flavonoid scaffold. In flavones and flavonols, the presence of a C2=C3 double bond together with a 4-keto group generates an extended conjugated system that promotes near-coplanarity of the A, B, and C rings. Homology modeling studies suggest that this rigid, planar architecture facilitates the insertion of flavonoid molecules into the aromatic hydrophobic cage of the OAT3 binding pocket, enabling strong π-π stacking interactions with key residues such as Tyr359 and Phe397 [9]. In contrast, flavanones and flavan-3-ols lacking the C2=C3 double bond adopt a more flexible, nonplanar C-ring conformation, which is generally associated with reduced transporter affinity [34,35].

Beyond planar stacking, substitution patterns on the B-ring play a decisive role in transporter recognition. Many high-affinity flavonoids, including quercetin and luteolin, contain an ortho-diphenolic (catechol) moiety at the 3′ and 4′ positions of the B-ring. Beyond conferring antioxidant and metal-chelating properties, this structural motif increases the topological polar surface area (tPSA) and provides multiple hydrogen-bond donor and acceptor sites, thereby enabling stable hydrogen-bond anchoring with polar residues within the OAT1 transport pathway [10,35].

Ultimately, among all determinants, ionization state represents the most critical feature for OAT recognition. While flavonoid aglycones typically exhibit weak acidity (pKa ~6–9) under physiological conditions, rapid Phase II metabolism in vivo introduces highly polar, negatively charged groups, converting them into potent organic anions [39,42]. Experimental evidence indicates that flavonoid monoglucuronide or sulfate conjugates display markedly higher affinity for OAT1 than their parent aglycones [12]. Accordingly, the combined motif of a hydrophobic aromatic backbone coupled with an anionic side chain is now regarded as the core pharmacophore underlying flavonoids’ preferential recognition as substrates or inhibitors of OAT1 and OAT3 [42,43].

### 3.3. In Vivo Metabolic Transformation: From Plant Glycosides to OAT Substrates

Although flavonoids predominantly exist as glycosides in plants, their circulating forms in vivo differ significantly from their native structures [34,35]. Following oral intake, flavonoid glycosides undergo deglycosylation mediated by small-intestinal brush-border enzymes and the gut microbiota, generating aglycones that can be absorbed by intestinal epithelial cells [41]. Once absorbed, these aglycones are rapidly subjected to extensive Phase II metabolism within the intestine and liver, primarily catalyzed by uridine 5′-diphospho-glucuronosyltransferases (UGTs) and sulfotransferases (SULTs). Flavonoids entering the systemic circulation exist predominantly as glucuronide and sulfate conjugates, whereas free aglycones are present only at negligible levels [39,41,42]. By converting lipophilic aglycones into highly hydrophilic, negatively charged organic anions, the Phase II conjugation process generates molecular entities that closely align with the canonical substrate profile of renal OAT1 and OAT3, thereby providing the physicochemical foundation for the efficient recognition and active secretion of flavonoid metabolites by these transporters [39,42].

Importantly, flavonoid conjugates should not be regarded as biologically inert. Certain glucuronide conjugates can be hydrolyzed by β-glucuronidase in target tissues, thereby regenerating active aglycones, while others may directly modulate intracellular signaling pathways. These findings challenge the traditional assumption that Phase II conjugation necessarily abolishes bioactivity and underscore the functional relevance of flavonoid metabolites in vivo [42,43].

### 3.4. Renal Disposition Characteristics and OAT-Mediated Nephroprotective Effects

The kidney represents the principal organ responsible for the elimination of flavonoid phase II metabolites, with OAT1/OAT3 constituting the critical rate-limiting determinants of their renal exposure and excretion. Accumulating transporter studies support a coherent mechanistic framework linking OAT-mediated transport, intrarenal accumulation, and nephroprotection.

Extensive experimental evidence demonstrates that flavonoids exert broad nephroprotective effects—including antioxidant, anti-inflammatory, anti-apoptotic, and anti-fibrotic actions—across diverse pathological models, such as acute kidney injury, drug-induced nephrotoxicity, diabetic nephropathy, and nephrotic syndrome [15,16,19,44,45,46,47]. Mechanistically, these protective effects involve three convergent pathways. First, flavonoids attenuate oxidative stress induced by nephrotoxins through reactive oxygen species scavenging and Nrf2–HO-1/NQO1 pathway activation [46]. Second, they suppress renal tubular inflammation by inhibiting NF-κB signaling, NLRP3 inflammasome activation, and downstream pro-inflammatory cytokines (e.g., TNF-α, IL-1β). Third, they mitigate tubulointerstitial fibrosis by regulating apoptotic pathways and profibrotic signaling networks, such as TGF-β/Smad and epithelial–mesenchymal transition [47].

Taken together, OAT1 and OAT3 function not merely as elimination pathways for flavonoid metabolites but also as intrarenal concentrators that facilitate their protective actions within renal tissue. This bidirectional interplay—whereby flavonoids modulate OAT activity through structure–activity relationships, and OATs in turn govern the intrarenal disposition and efficacy of flavonoids—forms the central conceptual foundation for the systematic SAR analyses presented in the subsequent sections of this review.

## 4. Research Status and Structure–Activity Relationships of Flavonoid-OAT1/OAT3 Interactions

### 4.1. Research Models and Methodological Basis

At present, investigations into flavonoid–OAT1/OAT3 interactions are predominantly conducted using heterologous expression systems. Classical experimental platforms include renal-derived cell lines (e.g., human embryonic kidney 293 (HEK293) and Lilly Laboratories cell-porcine kidney 1 (LLC-PK1)) stably or transiently expressing human OAT1 or OAT3, as well as the *Xenopus laevis* oocyte model [25,48,49]. Methodologically, these studies generally adopt two complementary strategies. First, inhibition-based assays are employed to determine inhibition constants (*K*_i_ or *IC*_50_) using prototypical probe substrates, such as para-aminohippurate (PAH) for OAT1 and estrone sulfate (ES) or the fluorescent substrate 6-carboxyfluorescein (6-CF) for OAT3 [10,50]. Second, direct transport studies utilize radiolabeled or fluorescently tagged flavonoids and their metabolites to quantify intracellular uptake kinetics (*K*_m_, *V*_max_), thereby confirming their substrate status [12,50]. In recent years, computer-aided drug design (CADD) approaches integrating homology modeling and quantitative structure–activity relationship (QSAR) analyses have been increasingly applied to elucidate the molecular determinants governing flavonoid recognition by OATs [9,11].

#### Critical Comparison of Computational Approaches: Docking, Dynamics, and Machine Learning

While molecular docking provides a valuable static snapshot of ligand-transporter interactions, elucidating the complex SAR of flavonoids with OATs increasingly relies on a multidimensional computational approach. Molecular docking is highly efficient for initial high-throughput screening and identifying putative binding orientations within the central cavity. However, because OAT1 and OAT3 are MFS transporters that undergo significant conformational transitions (the alternating access mechanism) during the transport cycle, static docking alone is often insufficient to capture dynamic “induced-fit” mechanisms.

To address this structural limitation, molecular dynamics (MD) simulations are employed. As demonstrated by Janaszkiewicz et al., MD simulations in lipid bilayers can reveal the temporal stability of the transporter-ligand complex and help capture transient conformational states that static models may overlook [19]. Furthermore, particularly for OAT3, where a high-resolution experimental structure remains elusive, ligand-based quantitative structure–activity relationship (QSAR) and machine learning models have become highly valuable. These data-driven approaches, such as those utilized by Ni et al. and Nigam et al., are effective for identifying key physicochemical predictors—such as topological polar surface area (tPSA) and lipophilic volume—across large compound libraries without relying on a rigid protein template [11,51]. Ultimately, the integration of these methodologies—combining structure-based dynamic simulations with ligand-based machine learning—represents a more comprehensive strategy for predicting flavonoid-OAT interactions.

In this review, to provide a foundational structural context for the OAT1 SAR discussion, a representative static docking visualization was employed. Briefly, the cryo-EM structure of human OAT1 (PDB: 9KKK) was prepared using standard protocols, and quercetin was docked into the central cavity using AutoDock 4 (version 1.5.6) with a Lamarckian Genetic Algorithm. The lowest binding energy pose was selected to theoretically visualize the spatial arrangement of key interacting residues. Protein–ligand interactions were profiled and visualized using the Protein–Ligand Interaction Profiler (PLIP) web server [52] and PyMOL (version 2.5).

### 4.2. Flavonoids as Inhibitors or Transport Substrates of OAT1/OAT3

Inhibition represents the most extensively documented interaction. Early studies by Hong et al. and An et al. demonstrated that multiple naturally occurring flavonoids, including morin, quercetin, and luteolin, inhibit OAT1- and OAT3-mediated uptake of probe substrates in a concentration-dependent manner, acting as high-affinity competitive inhibitors [10,50].

Importantly, increasing evidence indicates that flavonoids—particularly their phase II metabolites—also function as bona fide transport substrates. With respect to aglycones, Hong et al. reported significantly greater intracellular accumulation of morin in hOAT1-expressing cells compared with controls. Consistently, An et al. showed that luteolin and quercetin exhibit time-dependent uptake that is sensitive to probenecid inhibition, suggesting that certain aglycones themselves can be transported by OAT1 [10].

More physiologically relevant insights arise from studies on conjugated metabolites. Wong et al. demonstrated that glucuronide and sulfate conjugates of quercetin and genistein are high-affinity substrates for hOAT1 and hOAT3, often displaying greater transport efficiency than their parent aglycones and exhibiting isoform-specific selectivity [12,53].

Collectively, current evidence supports the consensus that flavonoids—particularly their predominant anionic metabolites in vivo—act simultaneously as transport substrates and potent competitive inhibitors of OAT1/OAT3. This dual role implies that flavonoids not only depend on OATs for renal elimination but can also substantially interfere with the renal disposition of co-administered OAT substrates through competitive occupancy of transporter binding sites [9].

### 4.3. Structure–Activity Relationships (SAR): From Molecular Structure to Transport Affinity

Based on the systematic screening studies of OAT1 and OAT3, several key structural determinants governing the inhibitory and transport activities of flavonoids have been identified [10,11].

#### 4.3.1. SAR Characteristics of OAT1: The “Stringent” Preference for Compact Planar Scaffolds

The substrate binding pocket of OAT1 is relatively small and imposes constraints on both molecular size and hydrophilicity [49].

Scaffold selectivity is evident, with flavones and flavonols exhibiting the strongest affinity for OAT1. The presence of a C2=C3 double bond and a 4-keto group in the C-ring maintains molecular planarity, facilitating insertion into the OAT1 active site [10].

Substituent effects further modulate affinity. Free aglycones consistently show higher activity than their glycosylated counterparts (e.g., rutin and phlorizin), indicating that deglycosylation is a prerequisite for effective OAT1 recognition. In addition, the presence of a 3′,4′-dihydroxyl (catechol) moiety on the B-ring generally enhances inhibitory potency [10,50]. This observation is supported by findings from Uwai et al., who reported that caffeic acid, bearing a similar catechol structure, markedly inhibits OAT1 activity [54].

**Figure 2:**
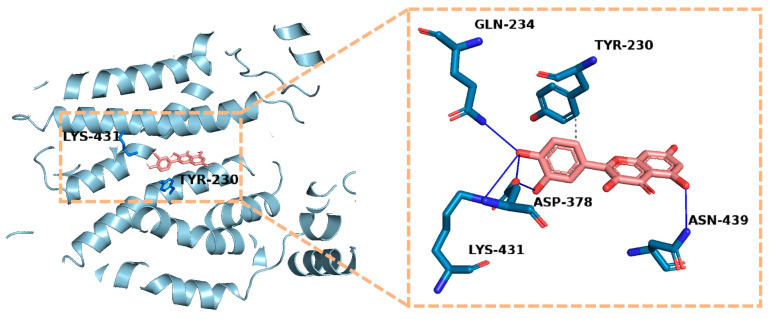
Molecular docking visualization of quercetin in the binding pocket of human OAT1. The computational model predicts the detailed interaction network within the central cavity (based on PDB: 9KKK). The overall OAT1 protein backbone is depicted in light blue, and the carbon atoms of the interacting amino acid residues are shown in blue. For the quercetin ligand, the carbon atoms are colored salmon, while oxygen and nitrogen atoms throughout the model are colored red and dark blue, respectively. The planar scaffold of quercetin is stabilized by hydrophobic and π–π stacking interactions (grey dashed lines) involving residues such as Tyr230. A dense hydrogen-bond network (blue solid lines) anchors the ligand to polar residues, notably Asp378, Lys431, Gln234, and Asn439. This theoretical visualization structurally supports the high-affinity recognition mechanism described in the SAR analysis. (OAT1, organic anion transporter 1; PDB, Protein Data Bank).

To theoretically visualize these SAR observations, molecular docking analysis was employed using the recently resolved cryo-EM structure of human OAT1 (PDB: 9KKK). As illustrated in the docking model (Figure 2, left panel for general view and right inset for detailed interaction network), the planar scaffold of quercetin is stabilized by hydrophobic and π–π stacking interactions (grey dashed lines) involving residues such as Tyr230. Crucially, the B-ring catechol moiety and other polar groups participate in a dense hydrogen-bond network involving Lys431, Asp378, Gln234, and Asn439. The specific functional roles and interaction types of these key residues are summarized in Table 1. This computational visualization aligns well with the experimental SAR data, highlighting the importance of specific electrostatic and hydrophobic anchoring in OAT1 substrate recognition.

#### 4.3.2. SAR Characteristics of OAT3: Tolerance for Bulky Glycosides and Conjugates

In contrast to OAT1, OAT3 possesses a more flexible substrate-binding pocket capable of accommodating molecules with extended aromatic scaffolds [49]. Accordingly, OAT3 exhibits broad-spectrum recognition, showing appreciable affinity for flavanones (e.g., naringenin), isoflavones, and methylated flavonoids, without a strict dependence on the C2=C3 double bond [11,50].

QSAR analyses by Ni et al. identified hydrogen bond acceptor distribution, lipophilic volume, and topological polar surface area (tPSA) as key predictors of OAT3 inhibitory activity. Flavonoid derivatives featuring larger hydrophobic substituents or specific polyhydroxylation patterns often display potent OAT3 inhibition (*IC*_50_ < 10 μM) [11]. Consistently, reviews by Chen et al. note that polyphenols such as epigallocatechin gallate (EGCG) inhibit OAT3 in a manner strongly associated with hydrophobicity [9].

## 5. Translational Applications Based on Structure–Activity Relationships: From Molecular Design to Systems Biology

Based on the experimental and mechanistic evidence summarized above, this section aims to distill generalizable principles governing flavonoid–OAT1/OAT3 interactions. Particular emphasis is placed on the recognition features of phase II metabolites, which represent the predominant in vivo forms of flavonoids, and on the translational implications of these principles for the rational design of nephroprotective agents and for the safety assessment of Traditional Chinese Medicine (TCM) preparations.

### 5.1. Navigating the Methodological Heterogeneity: A Comparative Perspective

The evaluation of flavonoid-OAT interactions relies heavily on the choice of in vitro models, each presenting distinct advantages and limitations (summarized in Table 2).

Transfected mammalian cells (e.g., HEK293, Chinese hamster ovary (CHO)) serve as the industry’s ‘gold standard’ due to their high throughput and single-transporter specificity, which are essential for establishing the precise SAR rules discussed in Section 3. However, a critical limitation for flavonoid research is their deficiency in Phase II metabolic enzymes (e.g., UGTs, SULTs). Since flavonoids undergo extensive conjugation in vivo, data derived solely from these metabolically incompetent models may overestimate the inhibitory potential of the parent aglycones while overlooking the effects of circulating metabolites.

In contrast, primary renal proximal tubule epithelial cells (RPTECs) offer a more physiologically relevant environment by retaining both transporter networks and metabolic competency. Yet, their application is restricted by rapid de-differentiation and inter-donor variability. Beyond metabolic competence, the choice of the experimental platform fundamentally influences the reported transporter affinities (e.g., *IC*_50_ and *K*_m_ values), thereby complicating cross-study interpretations. For instance, the apparent affinities of flavonoids often differ significantly between mammalian cell lines (e.g., HEK293) and the *Xenopus laevis* oocyte system. Such discrepancies can be largely attributed to differences in the cellular membrane lipid microenvironment—which intricately affects both the conformational dynamics of the transporter and the membrane partitioning of highly lipophilic flavonoids—as well as the differential expression of endogenous background transporters [55]. Furthermore, subtle variations in assay conditions across laboratories, particularly the presence and concentration of carrier proteins (e.g., bovine serum albumin) in the transport buffer, can markedly shift the measured *IC*_50_ values due to the extensive protein-binding characteristics of flavonoids [56]. Consequently, comparisons of apparent affinity values across different experimental models must be approached with caution. Instead, utilizing the relative ranking of affinities within a single standardized platform provides a much more reliable and robust basis for SAR interpretation. Therefore, a tiered strategy is recommended: utilizing transfected cells for initial high-throughput SAR screening, followed by validation in physiologically competent systems (e.g., RPTECs or physiologically based pharmacokinetic (PBPK) modeling) to bridge the gap between in vitro affinity and clinical safety.”

**Table 2:** Comparative assessment of in vitro models used for evaluating flavonoid–OAT interactions.

Model System	Advantages (Pros)	Limitations (Cons)	Applicability to Flavonoid Research	References
HEK293/CHO transfected cells	High throughput; Single-transporter specificity	Lack Phase II enzymes (UGTs, SULTs)	SAR screening	[3,10,11]
MDCK cells	Polarized transport studies	Limited transporter expression	Vectorial transport	[7,22]
Xenopus oocytes	Easy manipulation	Non-mammalian system	Kinetic studies	[3,57]
hRPTECs	Physiologically relevant; Retain metabolic capacity	Rapid de-differentiation; Donor variability	Validation studies	[3,57]
Kidney organoids	3D architecture; Multiple cell types	Immature phenotype; Cost	Advanced modeling	[3,57]

Abbreviations: CHO, Chinese hamster ovary cells; HEK293, Human embryonic kidney 293 cells; MDCK, Madin–Darby canine kidney cells; hRPTECs, Human renal proximal tubule epithelial cells; SAR, Structure–activity relationship; UGTs, UDP-glucuronosyltransferases; SULTs, Sulfotransferases.

### 5.2. General Pharmacophore Models and Selectivity Rules

**Figure 3:**
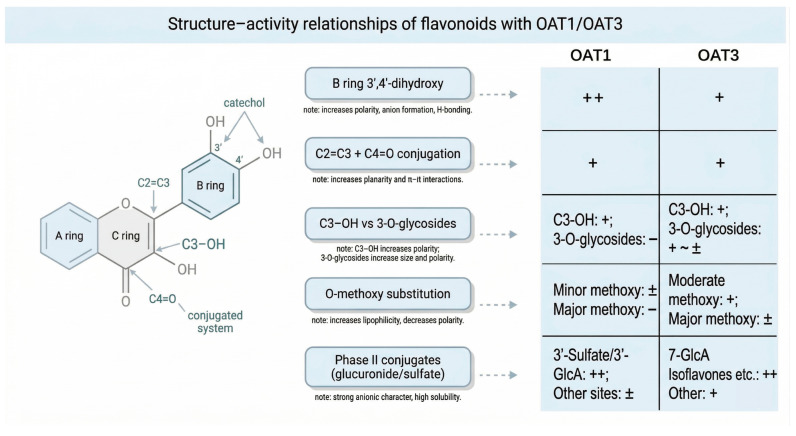
Schematic summary of key structure–activity relationships (SAR) of flavonoids interacting with renal OAT1/OAT3. “++” indicates strong inhibition or high-affinity substrate; “+” indicates moderate effect; “±” indicates weak effect or dependence on specific substitution patterns; “−” indicates significantly reduced or abolished effect. (SAR, structure–activity relationship; OAT1/3, organic anion transporter 1/3).

By integrating high-throughput screening data from previous studies [10,11,50], a consensus pharmacophore model for flavonoid recognition by OATs can be delineated (Figure 3).

Overall, OAT1 exhibits a stringent substrate specificity, preferentially recognizing molecules characterized by compact, planar scaffolds (e.g., flavones and flavonols), defined hydrogen-bond donor motifs (notably the B-ring 3′,4′-catechol moiety), and a distinct anionic center. In contrast, OAT3 displays a more permissive recognition profile. Attributable to its expansive hydrophobic pocket and capacity to accommodate diverse topological polar surface areas (tPSA), OAT3 interacts with bulkier or glycosylated flavonoids, including isoflavone glycosides and polymethoxylated flavones [11,58]. Representative inhibition constants (*IC*_50_) and the corresponding structural determinants are summarized in Table 3.

To systematically synthesize the diverse literature datasets and address the complex distinction between OAT1 and OAT3 selectivity, a consolidated, evidence-weighted comparative SAR framework is constructed. As summarized in Table 4, this framework explicitly highlights the distinct structural preferences, binding pocket architectures, and phase II conjugation tolerances between human OAT1 and OAT3.

### 5.3. Specific Recognition Patterns of Phase II Metabolites: The True In Vivo Substrates

Although in vitro experiments frequently employ flavonoid aglycones, pharmacokinetic evidence consistently demonstrates that glucuronide and sulfate conjugates dominate in systemic circulation and urinary excretion. Studies by Wong et al. and Järvinen et al. have revealed distinct and reproducible recognition patterns for these Phase II metabolites (Table 5) [12,42].

First, an anionization-driven affinity enhancement effect is observed. Conversion of neutral aglycones such as quercetin into 3′-sulfate or glucuronide conjugates introduces a strong negative charge, transforming them into high-affinity substrates for OAT1 and OAT3, with reported *K*_m_ values as low as 0.8 μM—often exceeding the affinity of the parent aglycone.

Second, site-selective conjugation critically determines transporter recognition. OAT1 is highly sensitive to the position of conjugation: quercetin 3′-conjugates retain high affinity, whereas modification at the 3- or 7-positions may abolish transport due to steric constraints [12]. In contrast, OAT3 efficiently transports 7-O-glucuronide-modified isoflavones, such as daidzein-7-GlcA [42].

Collectively, these findings indicate that aglycone-based SAR represents only a preliminary framework. In vivo, the motif of a retained hydrophobic aromatic scaffold combined with site-specific anionic conjugation constitutes the true “passport” enabling flavonoids to be recognized by OATs and to exert biological effects.

Furthermore, beyond flavonols, exploring diverse flavonoid subclasses is essential to fully appreciate the clinical implications of phase II metabolites. Recent pharmacokinetic studies demonstrate that the conjugation patterns of isoflavones (e.g., genistein and daidzein) and flavanones (e.g., hesperetin) yield subclass-specific glucuronide and sulfate metabolites that interact with OATs, influenced by their distinct spatial conformations [12,61]. This metabolic transformation fundamentally alters not only the pharmacokinetic behavior of flavonoids—facilitating systemic circulation and renal clearance—but also their intrinsic bioactivity. The traditional paradigm holds that phase II conjugation, particularly at active hydroxyl sites (such as the 3′,4′-catechol moiety), masks the pharmacophores essential for direct free-radical scavenging, thereby attenuating their antioxidant capacity. However, recent mechanistic reports reveal a more complex biological role. While direct in vitro antioxidant efficacy may be diminished, many circulating conjugated metabolites retain intracellular signaling capabilities, exhibiting significant anti-inflammatory and vascular-protective activities in vivo [62]. Moreover, these circulating phase II metabolites can act as systemic pro-drugs; upon reaching target tissues characterized by inflammation or tumor microenvironments, they can be locally deconjugated by tissue-specific β-glucuronidase to release the active parent aglycones [63]. Therefore, OAT-mediated handling of these conjugates is not merely an elimination pathway, but a critical regulatory mechanism that dictates the systemic residence time, target-site bioactivity, and tissue-specific delivery of these flavonoid pools.

### 5.4. Translational Application I: Strategies for Designing OAT-Targeted Nephroprotective Agents

The translation of in vitro SAR findings into in vivo nephroprotection follows a coherent “Structure–Affinity–Protection” logic. Our analysis reveals that the structural determinants of OAT affinity are effectively the predictors of nephroprotective potency. Flavonoids possessing a planar C2=C3 double bond and a B-ring catechol moiety (e.g., luteolin, quercetin) exhibit low-micromolar *IC*_50_ values against OAT1/OAT3. This high affinity allows them to competitively block the uptake of nephrotoxicants such as cisplatin and adefovir [10,13]. Phase II metabolic activation—specifically sulfation at the 3′-position—further amplifies this “shielding effect” by creating a high-affinity anionic pharmacophore that outcompetes toxic substrates for the transporter active site [12]. Conversely, glycosylation (e.g., rutin) or loss of planarity (e.g., naringenin) abolishes this competitive capacity, rendering such compounds ineffective as transporter-targeted protective agents.

From a translational standpoint, this competitive inhibition mechanism can be harnessed to mitigate drug-induced kidney injury (DIKI). OAT1/OAT3 are established gateways for the renal uptake of toxic compounds such as cisplatin, adefovir, and mercury [58,64]. Experimental evidence confirms the SAR-based predictions: Wong et al. demonstrated that quercetin-3′-O-sulfate, acting as a high-affinity substrate/inhibitor, dose-dependently reduces OAT1-mediated adefovir accumulation, resulting in marked attenuation of cytotoxicity [12]. In parallel, Neamatallah et al. showed that the natural polyphenol ellagic acid alleviates cisplatin-induced nephrotoxicity, at least in part, through modulation of OAT1/OAT3 expression [14]. Notably, promising candidates like apigenin offer dual protective mechanisms: reducing toxicant accumulation via OAT inhibition while simultaneously alleviating downstream renal histopathological injury via antioxidant pathways [13].

**Figure 4:**
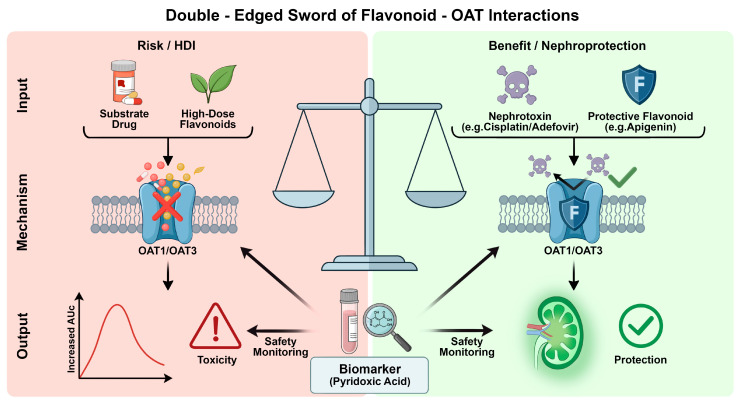
The “double-edged sword” effects of flavonoid-OAT interactions and the role of endogenous biomarkers. (**Left**) Risk: High-dose flavonoids inhibit OAT-mediated elimination of substrate drugs, leading to accumulation and toxicity (HDI). (**Right**) Benefit: Flavonoids competitively block the uptake of nephrotoxins (e.g., Cisplatin) into renal cells, providing nephroprotection. (**Bottom**) Biomarker: Endogenous substrates like pyridoxic acid serve as safety indicators for monitoring OAT modulation. (OAT, organic anion transporter; HDI, herb-drug interactions).

Based on these metabolite-centered SAR principles, ideal flavonoid-based nephroprotective agents should adhere to specific design strategies. First, a prodrug-oriented selection approach is recommended. Preference should be given to parent flavonoids that are rapidly converted in vivo into high-affinity anionic conjugates (e.g., quercetin 3′-sulfate), as this ensures the sustained generation of active metabolites that maintain competitive occupation of OATs at the basolateral membrane of renal tubules. Second, structural optimization should focus on the retention of key affinity-conferring motifs, such as the B-ring catechol structure or the C2=C3 double bond. These features should be combined with rational structural modifications that favor metabolism toward highly OAT-affine, rapidly excretable anionic forms, thereby achieving the dual benefits of sustained transporter competition and efficient systemic clearance. The comprehensive risk-benefit evaluation of this “double-edged sword” effect, along with the role of endogenous biomarkers, is conceptually illustrated in Figure 4.

#### Critical Evaluation of the Double-Edged Mechanism: Conflicting Reports and Limitations

While the “double-edged” mechanism provides a compelling framework, it must be critically evaluated against conflicting in vivo reports to ensure a balanced interpretation. First, several studies have demonstrated negligible in vivo OAT modulation despite potent in vitro inhibition. This discrepancy is largely attributed to the pharmacokinetic characteristics of flavonoids: their extensive plasma protein binding (often >95%) and rapid phase II metabolism substantially reduce the circulating free fraction of the active aglycone. Consequently, the free concentrations required to achieve clinically significant transporter inhibition or nephroprotection are frequently unattainable in vivo, rendering the expected herb–drug interactions or “shielding” effects unobservable in many trials [65].

Second, evidence of opposite (or paradoxical) effects further complicates this paradigm. While acute co-administration typically yields competitive inhibition of OATs (decreasing toxin uptake), prolonged exposure to certain flavonoids has been reported to induce contrasting effects at the transcriptional level. For example, chronic administration of flavonoids such as quercetin and rutin in hyperuricemic models has been shown to significantly upregulate the protein expression of OAT1 and OAT3 in the kidney to facilitate uric acid excretion [66,67]. In the context of drug-induced nephrotoxicity, such transporter upregulation could theoretically accelerate the renal accumulation of co-administered OAT-substrate nephrotoxins, thereby exacerbating kidney injury rather than preventing it. Furthermore, under specific microenvironmental conditions, the antioxidant properties of high-dose flavonoids can transition into pro-oxidant cytotoxicity, negating any transport-mediated protective benefits. Therefore, the double-edged model is not absolute and is highly contingent upon dosage regimens, protein binding, and chronic transcriptional regulation.

### 5.5. Translational Validation: Evidence from In Vivo Pharmacokinetic Studies

While the aforementioned SAR models provide a robust theoretical foundation, it is critical to validate these interactions using in vivo pharmacokinetic (PK) and clinical data. Recent in vivo studies have provided direct evidence that flavonoid–OAT interactions significantly alter systemic drug exposure. For instance, an in vivo PK study in rats demonstrated that the co-administration of flavonoid-rich herbal extracts (containing specific bi-flavones) significantly inhibited the OAT1-mediated renal clearance of the antiviral drug lamivudine. This transporter-mediated interaction resulted in a nearly 3-fold increase in the area under the plasma concentration-time curve (AUC) and a 1.87-fold increase in the maximum plasma concentration (*C*_max_) of the drug [68]. Similarly, epigallocatechin-3-gallate (EGCG), a predominant flavan-3-ol in green tea, was shown to potently inhibit both human and rat OAT1/OAT3, markedly reducing the in vivo renal elimination of OAT substrates and increasing their systemic retention in animal models [69]. Furthermore, from a systems biology perspective, metabolomic analyses of *Oat1* and *Oat3* knockout mice have confirmed that the systemic serum levels of various dietary flavonoids and their circulating metabolites are profoundly altered in the absence of these transporters, providing definitive in vivo proof of their physiological role in flavonoid disposition [5]. Although large-scale human clinical trials explicitly quantifying flavonoid–OAT drug interactions remain scarce, these in vivo animal PK data robustly validate the translational relevance of the proposed SAR framework and highlight the clinical risk of herb–drug interactions (HDIs) when high-dose flavonoid supplements are co-administered with narrow-therapeutic-index OAT substrates.

### 5.6. Translational Implications: Predicted Interaction Potential of Flavonoid-Containing TCM Preparations

The other side of this “double-edged” interaction paradigm involves the potential for transporter-mediated herb–drug interactions. With the expanding use of flavonoid-rich traditional Chinese medicine (TCM) preparations, modulation of OAT1/OAT3 activity represents a mechanistically plausible pathway for interaction.

#### 5.6.1. Predicted Interaction Potential Based on SAR Profiles

Preparations enriched in polymethoxylated flavones—identified as relatively potent OAT3 inhibitors in vitro—or formulated as high-concentration aglycone preparations may exhibit stronger predicted modulation of OAT3-mediated transport. Such modulation could theoretically influence the renal handling of OAT3-substrate drugs, including methotrexate and certain cephalosporins [58,60]. It should be emphasized that these considerations are derived from in vitro inhibition data and exposure assumptions rather than confirmed clinical interaction outcomes.

#### 5.6.2. Bridging SAR Insights to Complex Herbal Matrices

While structural analysis of individual flavonoids provides mechanistic insights, clinical exposure typically occurs through complex mixtures in foods or herbal preparations rather than isolated compounds. Therefore, the net effect on renal OAT activity likely reflects cumulative exposure to multiple flavonoid subclasses within these matrices. Based on the SAR principles discussed above, flavonoid-rich dietary and herbal sources can be tentatively grouped according to their predicted OAT modulation potential (Table 6). For example, preparations rich in polymethoxylated flavones (e.g., dried tangerine peel) may exhibit comparatively stronger predicted interaction potential than soy-based products rich in isoflavones, consistent with differences in scaffold lipophilicity and transporter binding tendencies.

#### 5.6.3. Considerations for Mechanism-Informed Safety Evaluation

From a translational perspective, incorporation of OAT inhibition profiling and assessment of phase II metabolic stability into TCM safety evaluation frameworks may facilitate mechanism-informed risk assessment. Particular attention may be given to compounds combining strong in vitro transporter inhibition with metabolic persistence and potential systemic exposure. However, the clinical relevance of these predictions requires validation through pharmacokinetic and interaction studies.

### 5.7. Systems Biology Perspective: OATs in the Gut–Liver–Kidney Axis

Beyond their established role in renal elimination, OAT1/OAT3 have been proposed, within the framework of the “Remote Sensing and Signaling” (RSS) theory, to participate in inter-organ metabolic communication [3,4]. Experimental and metabolomic studies in OAT-deficient models indicate that transporter activity influences the systemic handling of endogenous metabolites and selected diet-derived compounds, including flavonoid conjugates [4,5]. These findings suggest that OAT1/OAT3 contribute to the regulation of circulating metabolic intermediates and redox-related molecules, particularly within the gut–liver–kidney axis.

As flavonoids undergo extensive phase II metabolism and microbiota-mediated transformation, their interaction with OAT transporters may contribute to alterations in renal clearance and systemic distribution of these metabolites. While the broader RSS framework provides a systems-level perspective, current evidence primarily supports a role for OAT1/OAT3 in modulating specific endogenous and microbiome-derived solutes. Further quantitative studies are required to clarify the extent to which flavonoid-mediated OAT modulation influences systemic metabolic homeostasis.

## 6. Current Challenges and Future Perspectives

Although a foundational framework describing the structure–activity relationships (SARs) and translational implications of flavonoids with OAT1/OAT3 has been established, clinical translation remains influenced by two major methodological challenges: the in vitro–in vivo extrapolation (IVIVE) bias and mechanistic uncertainty surrounding substrate recognition. With recent advances in structural biology and systems pharmacology (2024–2025), future research may increasingly adopt structure-guided and biomarker-informed strategies to refine mechanistic understanding and improve predictive accuracy.

### 6.1. Current Knowledge Gaps in Flavonoid–OAT Interactions

Despite significant progress, several critical knowledge gaps remain that hinder the clinical translation of current SAR findings. First, while the cryo-EM structure of human OAT1 has been recently elucidated [17,18], a comparable high-resolution structure for OAT3 remains unavailable. Consequently, current OAT3 SAR models rely heavily on homology modeling, which significantly limits the precise atomistic understanding of how this transporter accommodates bulky, conjugated flavonoid metabolites. Furthermore, a massive, largely unexplored gap exists regarding the microbiome–metabolism–transporter axis. Current in vitro models predominantly focus on hepatic and intestinal phase II metabolites, such as glucuronides and sulfates. However, flavonoids are extensively metabolized by the gut microbiota into small phenolic acids prior to systemic absorption [70,71], and the specific interaction profiles of these microbial metabolites with OAT1 and OAT3 are not well defined. Finally, there is a significant translational disconnect regarding complex matrices. Most pharmacokinetic predictions are currently based on isolated single flavonoids, whereas in clinical practice, flavonoids are consumed as complex mixtures derived from herbal extracts or regular diets. How multiple flavonoids synergistically or antagonistically compete for OAT binding sites in vivo currently lacks comprehensive investigation and quantitative physiologically based pharmacokinetic (PBPK) modeling [72,73].

### 6.2. Translational Challenge: The “IVIVE Bias” and Metabolic Blind Spots

Most available SAR data derive from engineered cell models overexpressing OAT1 or OAT3. A notable limitation of these systems is their inability to replicate the human plasma environment, where flavonoids frequently exhibit >90% non-specific protein binding [74].

One major predictive pitfall is that *IC*_50_ values obtained from protein-free in vitro systems reflect total compound concentrations, whereas in vivo OAT-mediated transport is governed by the unbound fraction. Direct extrapolation from in vitro data may therefore overestimate clinical HDI potential. In addition, many experimental systems do not incorporate the coordinated interplay between metabolic enzymes (e.g., UGTs) and transporters, limiting their ability to reproduce integrated elimination dynamics observed in vivo [75].

Furthermore, this lack of metabolic competence creates significant “blind spots” in interaction assessments. Highly abundant phase II metabolites, such as quercetin-3′-O-sulfate, may exhibit distinct or even enhanced OAT affinity relative to their parent aglycones. Failure to account for these conjugated metabolites represents a significant source of uncertainty in current predictive models and may contribute to discrepancies between in vitro findings and in vivo observations.

### 6.3. Future Direction I: Structural Biology and Rational Design Opportunities

Historically, substrate recognition by OAT transporters relied largely on homology modeling and indirect biochemical inference. Building on the recently resolved cryo-EM structures of human OAT1 in both apo and inhibitor-bound conformations [17,18], substrate recognition can now be examined at higher resolution.

Analysis of these structures suggests that the OAT1 substrate-binding cavity comprises a unique aromatic hydrophobic cage capable of accommodating planar aromatic scaffolds, potentially through π-π stacking interactions and electrostatic complementarity.

Leveraging these models, structure-guided SAR analyses may allow systematic docking of flavonoid metabolites into experimentally resolved structures to explore interactions between key structural motifs (e.g., B-ring catechol groups or glycosylation patterns) and residues such as Arg466 and Tyr230. Such approaches may facilitate rational exploration of OAT1-selective modulators and provide a framework for investigating transporter-mediated nephroprotective strategies.

### 6.4. Future Direction II: Endogenous Biomarker-Guided PBPK Modeling

To enhance translational predictability, future evaluation strategies may transition from qualitative interaction alerts toward more quantitative modeling approaches. According to the “Remote Sensing and Signaling” (RSS) theory, OAT1/OAT3 regulate the systemic homeostasis of critical endogenous small molecules [3,4]. Consequently, perturbations in OAT function may be reflected not only in altered drug disposition but also in measurable shifts in endogenous metabolite profiles, providing a physiological basis for biomarker identification [27].

Physiologically based pharmacokinetic (PBPK) frameworks offer a “bottom-up” strategy for estimating systemic exposure changes under transporter modulation scenarios [28]. Importantly, pyridoxic acid (PDA), a major catabolite of vitamin B6, has been proposed as a specific endogenous probe substrate for OAT1/OAT3 activity [5,27]. Recent work by Thakur et al. (2025) demonstrated that the renal clearance of PDA is highly sensitive to OAT modulation, supporting its use as a mechanism-based indicator of altered transporter activity in vivo [5].

Model-based simulation of OAT-mediated PDA clearance could support estimation of potential exposure changes (e.g., area under the curve ratio (AUCR) shifts) of victim drugs in the presence of flavonoid-rich preparations [28]. While such biomarker-guided PBPK strategies remain under active development and require prospective validation, they may provide a quantitative framework to address long-standing uncertainties in IVIVE and transporter-mediated interaction prediction [5,27], potentially supporting probe-free safety assessment in complex exposure scenarios.

## 7. Conclusions

This review has synthesized current evidence regarding the molecular determinants, structure–activity relationships (SARs), and translational implications of flavonoid interactions with the renal organic anion transporters OAT1 and OAT3. Available data indicate that OAT1 and OAT3 function not only as renal elimination pathways but also as components of the broader “Remote Sensing and Signaling” network involved in systemic metabolic regulation.

Accumulating evidence suggests that flavonoid modulation of OAT-mediated transport is influenced by defined structural features, including the B-ring catechol moiety, the C-ring conjugated system, and specific phase II metabolic modifications. Such structure-dependent interactions underlie a context-dependent “double-edged” effect in translational settings: high-affinity flavonoids may competitively inhibit OAT function and alter drug disposition, while in certain scenarios they may also reduce renal tubular uptake of toxic substrates such as cisplatin.

Recent advances in structural biology and endogenous biomarker-guided PBPK modeling are improving mechanistic understanding of transporter–flavonoid interactions and may enhance quantitative prediction of transporter-mediated effects. Continued integration of structural, kinetic, and translational approaches will be important for refining the safety assessment of flavonoid-containing preparations and for guiding future exploration of transporter-targeted nephroprotective strategies.

## Figures and Tables

**Table 1:** Key amino acid residues governing substrate recognition in human OAT1 based on recent Cryo-EM structures and molecular docking.

Residue	Location	Interaction Type	Function
Lys431	TM10	H-bond	Catechol moiety anchoring
Asp378	TM7	H-bond	Substrate stabilization
Gln234	TM4	H-bond	Polar anchoring
Asn439	TM10	H-bond	Polar anchoring
Tyr230	TM4	Hydrophobic/π-π	Aromatic cage formation/Substrate stabilization

Note: The detailed interaction network was theoretically visualized via molecular docking (see Figure 2). (OAT1, organic anion transporter 1; cryo-EM, cryogenic electron microscopy; TM, transmembrane; H-bond, hydrogen bond).

**Table 3:** Summary of inhibitory activities (*IC*_50_) of representative flavonoids against human OAT1 and OAT3.

Compound	Structural Classification	Key Substitution Features	hOAT1 *IC*_50_ (μM)	hOAT3 *IC*_50_ (μM)	Selectivity/Activity Characteristics	References
Morin	Flavonol	B-ring 2’,4′-OH (Non-planar)	0.3–0.46	>50	Highly OAT1 selective; Potent inhibitor	[10,50]
Luteolin	Flavone	B-ring 3′,4′-OH (Catechol moiety)	0.47	~4.5	Dual OAT1/3 inhibition; Preference for OAT1	[10,13]
Apigenin	Flavone	B-ring 4′-OH (Non-catechol)	1.8	~2.9	Dual OAT1/3 inhibition; Slightly weaker activity than luteolin	[10,13]
Quercetin	Flavonol	B-ring 3′,4′-OH + C3-OH	6.43	2.83	Slightly higher affinity for OAT3 than OAT1; Dual inhibition	[10,50]
Fisetin	Flavonol	Lacks C5-OH	3.5	ND	Validated substrate of OAT1	[10]
Kaempferol	Flavonol	B-ring 4′-OH + C3-OH	9.4	5.8	Moderate dual inhibition	[10]
Rutin	Flavonol glycoside	C3-*O*-rutinoside (Bulky)	>100 (No inhibition)	>100	Loss of activity due to glycosylation (vs. Quercetin)	[10,50]
Quercitrin	Flavonol glycoside	C3-*O*-rhamnoside	>50	ND	Glycosylation significantly reduces OAT1 affinity	[10]
Naringenin	Flavanone	C2-C3 single bond (Non-planar)	26.8	6.5	Weaker activity than flavones/flavonols; Slightly stronger for OAT3	[10,50]
Genistein	Isoflavone	B-ring attached at C3	13.9	>50	Moderate/Weak inhibition	[10,12]
EGCG	Flavan-3-ol	Gallate ester group	3.08	3.86	Major tea polyphenol component; Dual inhibition	[9]

Note: ND indicates Not Determined or no data available. (OAT1/3, organic anion transporter 1/3).

**Table 4:** Comparative SAR Framework for Flavonoid Recognition by Renal OAT1 and OAT3.

Structural/Functional Feature	Human OAT1	Human OAT3
Binding Pocket Architecture	Smaller, relatively constrained central cavity; strict steric limitations [59].	Expansive, highly flexible hydrophobic pocket; highly accommodating [11,59].
Preferred Core Scaffold	Strict requirement for compact, planar scaffolds (e.g., flavones, flavonols) [60].	High tolerance for non-planar, bulky, and flexible scaffolds (e.g., flavanones) [51].
C-ring Conjugation (C2=C3)	Highly critical for maintaining coplanarity and high-affinity π–π stacking [10,50].	Less critical; readily accommodates C2-C3 single bonds [60].
B-ring Substitution	Strongly favors 3′,4′-catechol moiety for dense hydrogen-bond networks [10,50].	Tolerates diverse substitutions, including substantial methoxy (-OCH_3_) groups [11].
Phase II Conjugation Preference	Highly selective. Prefers small, site-specific conjugates (e.g., 3′-sulfates). Steric clash with bulky 3- or 7-O-glucuronides [59].	Broadly accommodating. Readily transports bulky 7-O-glucuronides and other large phase II metabolites [12,42].
General Interaction Role	Primary target for unglycosylated, planar, highly polar anionic metabolites [15].	Primary target for bulky, polymethoxylated, or extensively conjugated flavonoids [51].

Note: Structural preferences summarized in this table are derived from integrated evidence including inhibition assays [10,11,49], transport kinetics studies [12,41], cryo-EM structural analyses [17,18,58], and computational modeling [11,50]. (OAT1/3, organic anion transporter 1/3.)

**Table 5:** Interaction modes and kinetic parameters of representative flavonoid Phase II metabolites with human OAT1 and OAT3.

Metabolite Name	Parent Compound	OAT1 *K*_m_ (μM)	OAT3 *K*_m_ (μM)	Mode of Action	References
Quercetin-3′-sulfate	Quercetin	0.8 ± 0.1	1.2 ± 0.2	Substrate/Inhibitor	[12,42]
Quercetin-3-glucuronide	Quercetin	>50	2.5 ± 0.4	Substrate	[12]
Quercetin-3′-glucuronide	Quercetin	ND	3.8 ± 0.6	Substrate	[12,53]
Genistein-7-glucuronide	Genistein	ND	4.2 ± 0.7	Substrate	[9,12,42]
Daidzein-7-glucuronide	Daidzein	ND	5.6 ± 0.9	Substrate	[9,12,42]
Kaempferol-3-glucuronide	Kaempferol	1.5 ± 0.2	2.1 ± 0.3	Substrate	[9]
Luteolin-3′-sulfate	Luteolin	0.8 ± 0.1	1.2 ± 0.2	Substrate/Inhibitor	[10,42]

Note: ND indicates Not Determined or no data available. *K*_m_ values represent mean ± SD. (OAT1/3, organic anion transporter 1/3).

**Table 6:** Predicted OAT1/OAT3 modulation potential of representative flavonoid-rich dietary sources and herbal preparations based on in vitro inhibition profiles and exposure considerations.

Source	Major Flavonoid Class	Reported OAT1/OAT3 Interaction Profile (In Vitro)	Key Co-Medication Considerations (Examples)	Predicted Interaction Potential *
Citrus peel (Chenpi)	Polymethoxylated flavones	Strong OAT3 inhibition [11,58]	Potential retention of OAT3 substrates (e.g., MTX, cephalosporins)	High
Green tea	Catechins (e.g., EGCG)	Moderate OAT3 inhibition [9]	Potential interference with clearance at high systemic exposure	Moderate
Onion, apple	Quercetin derivatives	OAT1/OAT3 substrate/inhibitor [12,13]	Nephroprotection; Low HDI risk	Low
Soy products	Isoflavones	Moderate OAT3 substrate [42]	Low HDI risk; Efficient excretion	Low
Ginkgo biloba	Flavonol glycosides	Weak OAT inhibition [3,10]	Minimal interaction expected	Low
*Scutellaria baicalensis*	Baicalin (and aglycones)	OAT3 substrate/inhibitor [9,60]	Potential nephroprotection	Low-Moderate

* Predicted interaction potential represents a qualitative categorization derived from reported in vitro OAT1/OAT3 interaction profiles and exposure considerations. These categories do not represent confirmed clinical interaction risk or therapeutic benefit and require validation in pharmacokinetic and clinical studies. Abbreviations: HDI, herb–drug interaction; MTX, methotrexate; EGCG, epigallocatechin gallate.

## Data Availability

No new data were created or analyzed in this study. Data sharing is not applicable.

## Abbreviations

The following abbreviations are used in this manuscript:

6-CF	6-Carboxyfluorescein
α-KG	α-Ketoglutarate
AKI	Acute kidney injury
CADD	Computer-aided drug design
CHO	Chinese hamster ovary cells
ciPTEC	Conditionally immortalized human proximal tubule epithelial cells
CKD	Chronic kidney disease
DDI	Drug–drug interactions
DIKI	Drug-induced kidney injury
EGCG	Epigallocatechin gallate
EMT	Epithelial–mesenchymal transition
ES	Estrone sulfate
HDI	Herb–drug interactions
HEK293	Human embryonic kidney 293 cells
hRPTECs	Human renal proximal tubule epithelial cells
IVIVE	In vitro–in vivo extrapolation
LGA	Lamarckian Genetic Algorithm
OAT1	Organic anion transporter 1
OAT3	Organic anion transporter 3
PAH	Para-aminohippurate
PBPK	Physiologically based pharmacokinetic
PDA	Pyridoxic acid
QSAR	Quantitative structure–activity relationship
RSS	Remote Sensing and Signaling
SAR	Structure–activity relationship
SULTs	Sulfotransferases
TCM	Traditional Chinese medicine
TM	Transmembrane
tPSA	Topological polar surface area
UGTs	Uridine 5′-diphospho-glucuronosyltransferases
